# Handling of the Covid-19 Pandemic and Its Effects on Bariatric Surgical Practice: Analysis of GENEVA Study Database

**DOI:** 10.1007/s11695-022-06267-7

**Published:** 2022-10-25

**Authors:** Rishi Singhal, Tom Wiggins, Sjaak Pouwels, Yashasvi Rajeev, Brijesh Madhok, Wasim Hanif, Abd A. Tahrani, Yitka Graham, Christian Ludwig, Kamal Mahawar, On behalf of the GENEVA Collaborators, Miguel Lamota, Salah M. Raslan, Aziz Sumer, Surendra Ugale, Radwan Kassir, Ahmet Z. Balta, Krishnamohan Yarlagadda, Marcelo L. Fage, Francisco Aguilar Espinosa, Aloy J. Mukherjee, Pedro R. Martinez Duartez, Digvijaysingh Bedi, Mahir M. Ozmen, Mohammad Kermansaravi, Edoardo Baldini, Mahendra Narwaria, Osama Mohammed Murshid, Abou-Mrad Adel, Chirag Parikh, Christian O. Ramirez-Serrano, Francesco Martini, Randeep Wadhawan, Ronnal P. Vargas, Francesco Pizza, Sergio Carandina, Mehmet Celal Kizilkaya, Miroslav Ilić, Patricio A. Lamoza, Tuna Bilecik, Marcelo C. Torres, Cesar Guevara, Jose Eduardo Garcia-Flores, Nasser Sakran, Sebastian Arana-Garza, Manish Khaitan, Faruk Karateke, Victor Valenti, Nicola Tartaglia, Nandakishore Dukkipati, Sonja Chiappetta, Mario Musella, Manuel G. Carvalho, Enrico Pinotti, Arun Prasad, Kamran Shah, Efstratia Baili, Syed Imran Abbas, Carlo Nagliati, Octávio Viveiros, Rui J. S. Ribeiro, Luigi Angrisani, Ian S. Soriano, Ahmed Chakib Abbadi, Nilton T. Kawahara, P. Praveen Raj, Ghulam Siddiq, Hany A. Balamoun, Carlos Vaz, Aparna Govil Bhasker, Jacques Himpens, Ajjana Techagumpuch, Apoorv Shrivastava, Mahidhar Valeti, Bernard Bokobza, Ahmad Bashir, Salvatore Avallone, Hugues Sebbag, Miguel Angel Escarti Uso, Agustin E. Rodriguez, Diego Awruch, Camilo Ortiz Silva, Amador Garcia Ruiz De Gordejuela, Chih-Kun Huang, Emilio Manno, Elena Ruiz-Ucar, Jose M. Balibrea, Steven Paul Marcoen, Cuneyt Kirkil, Almantas Maleckas, Dang Tuan Pham, Eric J. Hazebroek, Waleed Al-Khyatt, Tigran Poghosyan, Julian W. Mall, Rajat Goel, Patrick Noel, Vivek Bindal, Gaurav Prasad, Oscar M. Gomez Davila, Lilian Kow, Marc Focquet, Taryel Omerov, Athanasios Pantelis, Hércio Azevedo De Vasconcelos Cunha, Carlos Zerrweck, Viore Dejeu, Safwan A. Taha, Yannko G. Dominguez, Catalin Copaescu, Adriano Ribeiro Meyer Pflug, Fernando J. Martinez-Ubieto, Antonella Usai, Girish Kumar Juneja, Mahmoud Moustafa Basho, Nahum Beglaibter, Tadeja Pintar, Neslihan Ağbaba, Marco Anselmino, Anders Thorell, Ozan Şen, Tom Wiggins, Nelson F. Trelles, Gurdal Oren, Andrew G. N. Robertson, Elias Chousleb Mizrahi, Gustavo Sevá-Pereira, Fabio A. Carvalho, Ahmed Khalil Salman, Giovanni Dapri, Prashant H. Salvi, Murat Uston, Amir Hosein Davarpana ah Jazi, Abdollah Zandi, Mustafa I. Allouch, Camilo Boza, Carlos Esquivel, Miguel A. Carbajo, Maaz Ul Hassan, Adrian Augusto Graniel Diaz, Mohamad Hayssam Elfawal, Jose Vicente Ferrer, Davide Mazza, Stefano Olmi, Vandana Soni, Matteo Uccelli, Gregory E. Jones, Lakshmi S. Kona, Daniel Cottam, Bekkhan B. Khatsiev, Mauricio Zuluaga Zuluaga, Khaled Gawdat, Heitor P. Povoas, Piotr Major, Hazem Al-Momani, Marina Kurian, Fabio Cesare Campanile, David Hazzan, Antone Muneer Alhallak, Grzegorz Józef Kowalski, Krzysztof Kaseja, Markos Daskalakis, Asnat Raziel, Konstantinos Albanopoulos, Alexandros Charalabopoulos, Guido Jutten, Parag G. Patel, Anmol Ahuja, Tarun Mittal, Asim Shabbir, Magan Mehrotra, Enrico Facchiano, Andre Morrell, Antonio J. Torres, Ronald Liem, Terry L. Simpson, Almino C. Ramos, Mazen Takieddine, Sandeep Aggarwal, Pradeep Chowbey, Luigi Piazza, Alen Pajtak, Mohamad Aznan Shuhaili, Zdenko Boras, Juan S. Azagra, Mohamed Gamal Qassem, Mohey R. Elbanna, Abdulmajid Ali, Rutger Franken, Dimitri Pournaras, Sami Mansour, Nestor Apáez Araujo, Abraham Krikhely, Chetan Parmar, Marcelo Lo, Hqbib Ajami, Rajanikanth Yarram, Hasan Kais, Omer Al-Taan, Michael W. Hii, Francisco J. Barrera Rodriguez, Hosam M. Elghadban, Jorge Jpc Pérez Cruz, Salvador Ramirez, André Lázaro, Manel Riera, Sherif Awad, Guilhermino N. S. Neto, Mauricio E. Valencia A, Juan C. Olivares, Juan A. Altuve, Jitesh Parmar, Ricardo V. Cohen, Sergio Verboonen, Maurizio De Luca, Heath J. Antoine, Yangel Núñez Santana, Jhon C. Carrasco Flores, Ricardo Cuellar Tamez, Gilberto Ungson, Paulina Salminen, Evren Dilektasli, Luciano Antozzi, Hussam Z. Adi, Adolfo Leyva-Alvizo, Sandra Viviana Andino, Rey J. Romero, Nasir Nizami, Monika Proczko-Stepaniak, Marleen Romeijn, Isaac Walker Abreu, Mark Peter, Salena M. Ward, Ricardo Nassar, Hany Mohamed Abdulrahman Aboshanab, Juan F. Ortega Puy, Mohammed Khalid Mirza Gari, Wah Yang, Franco Favretti, Jon A. Kristinsson, Moataz M. Bashah, Luis Flávio Vilela De Mesquita, Felipe J. Cantu, Halit Eren Taskin, Jesus Gonzalez, Peter Lamb, Cristian E. Boru, Abdulzahra Hussain, Bilal Alkhaffaf, P. S. Jambulingam, Chek H. Tog, Jorge D. Picardo, Aleksandr Neimark, Basil J. Ammori, Zhiyong Dong, Ubaldo H. Garcia Trujillo, Laurent Abram Layani, Vincenzo Salsano, Avinash Tank, Bruno Zilberstein, Denis Pajecki, Arin K. Saha, Talat Al Shaban, Ersun Topal, Donald Van Der Fraenen, Manuel Enrique Jimenez Amin, Fernando P. Galaz, Nael Z. Abdo, Abbas Abdel Rahman Mohamed, Luis Poggi, Hüseyin Çiyiltepe, Cacio Ricardo Wietzycoski, Giuseppe G. S. Scalera, Ramen Goel, Newton Teixeira Santos, Aatif Inam, Esther Mans, Mohammad Altarawni, Mohammed Al Hadad, Abdelhadi Mejdane, Abdul Aziz Saleh Mhanna, Santiago Martin, Murat Akbaba, Shahzad Alam Shah, Dieter Birk, Md Tanveer Adil, Máximo Max Torres, Haitham Mostafa Elmaleh, Karl Miller, Kirubakaran Malapan, Hikmat Matar, Ravikrishna Mamidanna, Ahmad A. Gudal, Emad A. Aljohani, Jose Luis Estrada, Felipe E. Fiolo, Mohd Nizam Md Hashim, Manuel-Rodrigo Prieto-Aldape, Mourad Niazi, Ricardo X. Cuellar Tamez, Jerome Dargent, Obaid M. Alharbi, Abhishek Katakwar, Feras Dalati, Sharad Sharma, Tarig A. Samarkandy, Miguel Angel M. F. Farina Del Rio, Surrendar Dawani, Maria-Teresa Van Der Merwe, Marcos Leão P. Vilas-Boas, Alaa Abbass Moustafa, Oleg Dukhno, Ahmed Ahmed, Foolad Eghbali, Samik K. Bandyopadhyay, Amir Ul Haq Khan, Alan GK Li, Matyas Fehervari, Eduardo Silva, Marcos Kostalas, Tamir Salih, Hosam Hamed, Roel Bolckmans, Bassem Amr, Richard Welbourn, Jose Arturo Meneses Cervantes, Vinod Menon, Bernardo Marzano, Manuel Garcia Garza, Sumit Talwar, Jose Alfredo Jimenez, Jaime R. Ramos-Kelly, Rami Lutfi, Farah A. Husain, Helen M. Heneghan, Kirtik Patel, Maurílio Ribeiro Junior, Fabio Viegas, Manuel Avalos-Avalos, Zubaidah Nor Hanipah, Rob Snoekx, Camilo A. Diaz Rincon, Rodrigo Aceves, Muayad Fadhel, Steven A. Cahalan, Dhafer M. Jasim, Mohammed Salim Al-Hamadani, Mohannad Kamel Albermani, Hussein Saleh Ali, Wissam Jaafar Altaee, Hamid Dawood Almussawi, Antonio S.B. Silva, Ammar A. Atra, Abdulaziz Abood Majeed, Ahmed Nasser Al-Turfi, Omar Salem Alomar, George Kalogeropoulos, Ivaylo Georgiev Tzvetkov, Rana Manindra Rajneesh, Haris Khwaja, Diego Foschi, Georges Nabih Al Hajj, Ahmad Assalia, Fadil Khaleal, Maria Solovyeva, Abdou Abdalla Ali Salem, Hany Takla, Haider A. Alshurafa, Nazim Alrifai, Andres Muñoz-Mora, Gabriel Martinez De Aragon, Victor V. Diaconu, Naif A. Alenazi, Mehmet Kaplan, Paulo C. Grippa, Peter D. Nottle, Luis Antonio C. Fonseca, Roger C. Luciani, Michael L. Talbot, Yun Chan Park, Eduardo Nacur Silva, Giovanni Merola, Vikrant Sharma, Abdelrahman M. Elghandour, Estuardo J. Behrens, Alistair Sharples, Jose M. Pestana, Jeronimo Monterrubio, Eduardo Lemos De Souza Bastos, Naser Saleh Alalwani, Diya Aldeen Mohammed, Heidi Louise Kathrein, Francois N. Schutte, Adrian Sava, Mª De Los Angeles Mayo-Ossorio, Dick A. Manrique, Shahab Shahabi, Cem EMIR Guldogan, Daniel Gärtner, Rachid KSAN Ksantini, Etienne Boutry, Guilherme S. Mazzini, Karl P. Rheinwalt, Alberto Pagan, Oral Ospanov, Sukhvinder Singh Saggu, Saud AES. Alsubaie, Maciej Walędziak, Basmah Fallatah, Andreas Edenberg, Mohammed Abdullah, Neil R. Floch, Johnny Stewart, Daniel V. Timofte, Aram E. Jawed, Amit Bhambri, Mohamed Hany, Sapan A. Jain, Ioannis Terzis, Luis Level, Mohamad Abdulkader Al Sayyad, Hassan Ahmed, Michael Devadas, Antonio Cláudio Jamel Coelho, Shashank S. Shah, Rodrigo J. Anacona C., Alexander B. Palacios, Diyaree Nihad Ismael, Usama Iqbal, Héctor R. Herrera, David Goitein, Reynaldo M. Quinino, Georgios Spiliopoulos, Pablo Pjz Zambrana, Rodrigo Villagran, Ahmad Ghazal, Francesco Frattini, Marco Battistoni, Konstantinos Stamou, Zsolt Bodnar, Hüseyin Sinan, Vijaya L. Nirujogi, Osama Taha, Songhao Hu, Mohammad Eid M. Mahfouz, L. Ulas Biter, Hamza Ibrahim, Lynz Jordan, January Hill, Mohammed Mustafa Hassan Mohammed, Luis X. Armijos, Ramon Vilallonga, Luis Alberto Zabala Salazar, Jerome F. Schrapps, Khalid Al Amri, Guillermo J. Muzio, Abdulmenem Yahya Abualsel, Marina Kurian, Lukasz Szczerbinski, Carlos M. Trindade, Ahmed Forieg, Fallon Schwoch, Laurent Genser, Ahmed Osman, Mariano De Almeida Menezes, Halil Özgüç, Hercio A. V. Cunha, Mohamed Saïd Sbaï Idrissi, Barış Gülcü, Alessandro Contine, Lucas Felix Rossi, Arda Isik, Omar A. Khan, Luiz Gustavo De Oliveira E. Silva, Mehmet Kadir Bartın, Samet Yardımcı, Erkan Yardimci, E. Paulo Pinto, Ibrahim Abdelhamid Hassan, Chee Loong Yeap, Rafael Arias, Ahmed H. Hamouda, Evelyn A. Dorado, Celso Simoneti, Peter Vasas, Luca Paolino, Roberto Cisneros De Ajuria, Vincenzo Borrelli, Adeel A. Shamim, Miguel F. Herrera, Julio Galindo Alvarez, Wei Jin Wong, Shalvin R. Prasad, Sherif Aly, Fatih Can Karaca, Sylvia Weiner, Mehmet Altug Kazak, Manish Motwani, Çağrı Büyükkasap, Andrea Rizzi, Samiullah Khan Niazi, Madhi Hashim Alatrakhiam, Nikolaos Pararas, Aini F. Ibrahim, Dali Youssef, Joao Caetano Marchesini, Jose-Maria V. Correia-Neves, Aditi Shreekumar, Ibrahim Elwardany, Semra Demirli Atici, Javier Lorenzo Pérez, Kin Cheung Ng, Christine Stier, Mohammed Hany Ashour, Haitham M. Elmaleh, Carlos A. S. Madalosso, Peter Vasas, Salah Raslan

**Affiliations:** 1 Upper GI Unit, Birmingham Heartlands Hospital, University Hospitals Birmingham NHS Foundation Trust, Birmingham, B9 5SS UK; 2 Department of Surgery, Agaplesion Bethanien Krankenhaus, Frankfurt am Main , Hessen Germany; 3 Department of Intensive Care Medicine, Elisabeth-Tweesteden Hospital, Tilburg, The Netherlands; 4 Pediatric Accidents and Emergencies Department, Northwick Park Hospital, London Northwest University Healthcare NHS Trust, London, UK; 5 East Midlands Bariatric and Metabolic Institute, University Hospital of Derby and Burton NHS Foundation Trust, Derby, UK; 6 Diabetes Department, University Hospital Birmingham UK and Birmingham City University, Birmingham, UK; 7 Centre for Endocrinology, Diabetes and Metabolism (CEDAM), Birmingham Health Partners, Birmingham, UK; 8 Clinical Drug Development, Novo Nordisk, Søborg, Denmark; 9 Faculty of Health Sciences and Wellbeing, University of Sunderland, Sunderland, UK; 10 Facultad de Psycologia, Universidad Anahuac Mexico, Mexico City, Mexico; 11 Bariatric Unit, South Tyneside and Sunderland NHS Trust, Sunderland, UK; 12 Institute of Metabolism and Systems Research, College of Medical and Dental Sciences, University of Birmingham, Birmingham, UK

**Keywords:** Bariatric surgery, COVID-19, SARS-CoV-2, GENEVA, Pandemic, Public health, Global health

## Abstract

**Background:**

The coronavirus disease 2019 (COVID-19) pandemic led to a worldwide suspension of bariatric and metabolic surgery (BMS) services. The current study analyses data on patterns of service delivery, recovery of practices, and protective measures taken during the COVID-19 pandemic by bariatric teams.

**Materials and Methods:**

The current study is a subset analysis of the GENEVA study which was an international cohort study between 01/05/2020 and 31/10/2020. Data were specifically analysed regarding the timing of BMS suspension, patterns of service recovery, and precautionary measures deployed.

**Results:**

A total of 527 surgeons from 439 hospitals in 64 countries submitted data regarding their practices and handling of the pandemic. Smaller hospitals (with less than 200 beds) were able to restart BMS programmes more rapidly (time to BMS restart 60.8 ± 38.9 days) than larger institutions (over 2000 beds) (81.3 ± 30.5 days) (*p* = 0.032). There was a significant difference in the time interval between cessation/reduction and restart of bariatric services between government-funded practices (97.1 ± 76.2 days), combination practices (84.4 ± 47.9 days), and private practices (58.5 ± 38.3 days) (*p* < 0.001).

Precautionary measures adopted included patient segregation, utilisation of personal protective equipment, and preoperative testing. Following service recovery, 40% of the surgeons operated with a reduced capacity. Twenty-two percent gave priority to long waiters, 15.4% gave priority to uncontrolled diabetics, and 7.6% prioritised patients requiring organ transplantation.

**Conclusion:**

This study provides global, real-world data regarding the recovery of BMS services following the COVID-19 pandemic.

**Graphical abstract:**

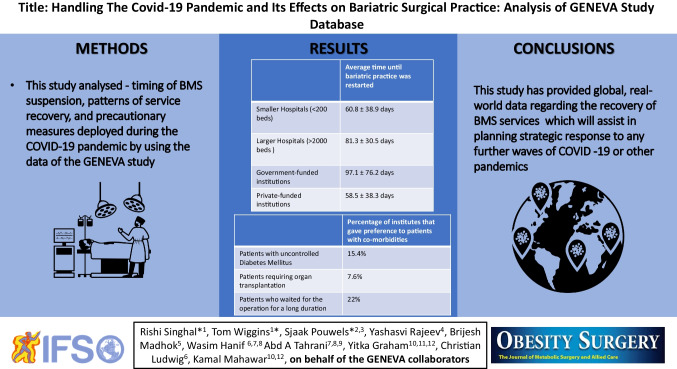

**Supplementary Information:**

The online version contains supplementary material available at 10.1007/s11695-022-06267-7.

## Introduction

The COVID-19 pandemic has had a devastating impact on healthcare services worldwide. During the early stages of the pandemic, the majority of elective surgical services needed to be paused [1]. There was evidence that recent infection with severe acute respiratory syndrome coronavirus 2 (SARS-CoV-2) would significantly increase risks associated with surgery [2]. With regards to bariatric and metabolic surgery (BMS), the International Federation for the Surgery of Obesity and Metabolic Disorder recommended all non-emergency bariatric surgery be suspended during the initial phase of the COVID-19 pandemic [3]. Studies confirm that BMS services were almost entirely paused during this phase of the pandemic [4, 5].

The COVID-19 pandemic arrived on top of a worsening obesity pandemic [6]. Patients with obesity are at increased risk of developing severe COVID-19-related disease, and the COVID-19 pandemic has had a further adverse impact on obesity rates due to the repeated periods of ‘lockdown’ and disruption to both medical and surgical weight management services worldwide along with changes in lifestyle leading to less physical activity and more calorie consumption [7, 8].

With the development of vaccines and improved medical therapeutics for COVID-19, we may have put the worst of the pandemic behind us but the critical question at this stage is what lessons can be learnt from the international response to the COVID-19 pandemic and how this may influence the strategic response to recovery of BMS services, further COVID-19 waves, or other global pandemics. Concerning BMS this should have a particular focus on how it will be possible to maintain services or recover swiftly in such eventualities.

The current study has utilised hospital-level data collected as part of the GENEVA study [9–11] to analyse patterns of service delivery, recovery of practices, and protective measures taken during the COVID-19 pandemic. The aim was to identify the strategies deployed during the recovery of BMS services following COVID-19.

## Methods

The current study is a subset analysis of the GENEVA study. The GENEVA study was a global, multicentre, observational study of BMS (elective primary, elective revisional, and emergency) performed between 1/05/2020 and 31/10/2020 in the adult (≥ 18 years). Detailed methods have been published previously [9, 10]. This study was registered as a multinational audit at the host institution [blinded] with registration number 5197. The hospital-level data collected detailed and anonymised information about surgeons, their respective bariatric surgical centres, the effect and handling of the pandemic at their centres, local perioperative COVID-19 safety protocols, and the timelines for the pandemic in their respective hospitals along with the chronology of the hospital response. Data for time points were collected to understand how different practices handled the pandemic. These time points included the first case diagnosed in the city, the first case admitted to the hospital, the peak in hospital admissions, stoppage, and subsequent restart of services. Only practices with all valid time points were considered for this part of the analysis. Data on these aspects were captured from participating surgeons through 28 questions (Appendix 1).

### Statistical Analysis

Continuous data were expressed as mean with standard deviation (SD). Data distribution was tested using the Kolmogorov–Smirnov test. Categorical data were presented as a number with percentages where appropriate. Patient characteristics and outcomes were compared using a Mann–Whitney *U*-test for continuous variables and a chi-square test for categorical variables.

A comparison of means was performed using the Kruskal–Wallis test. For these models, only cases with a complete data set were used. All tests were two-sided, and a *p*-value < 0.05 was considered statistically significant. All data were analysed using a statistical software package (SPSS™ Inc., version 24, Chicago, IL, USA).

**Fig. 1:**
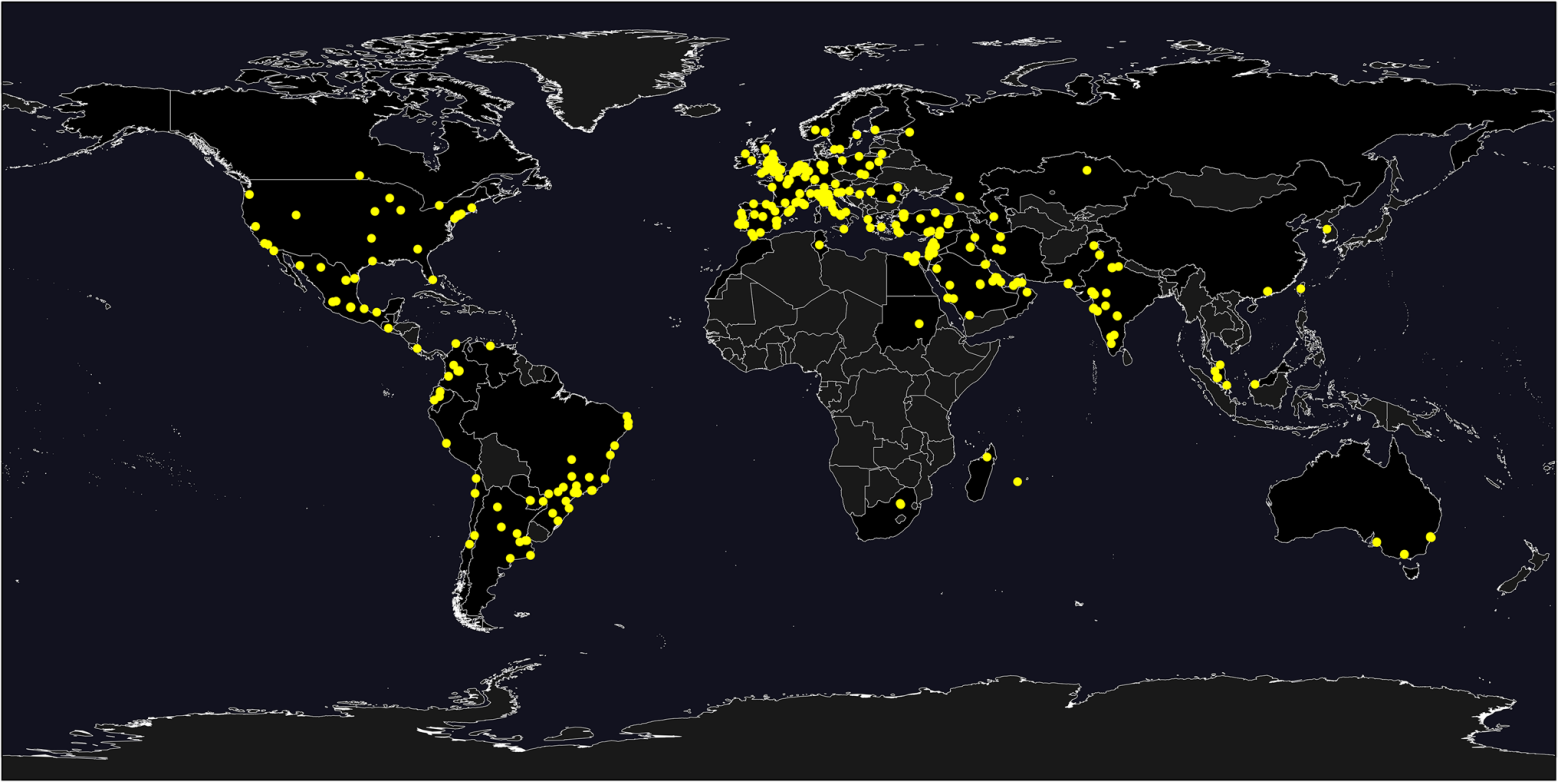
Overview of the geographical distribution of hospitals included in this study

**Fig. 2:**
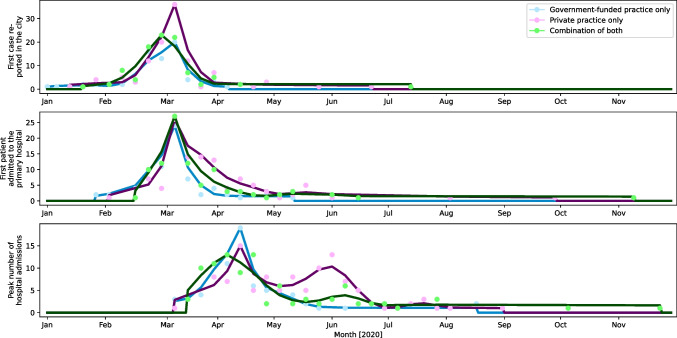
Timeline of the first case diagnosed in the city, the first case admitted to the primary hospital, and the peak number of hospital admissions according to the practice type

**Fig. 3:**
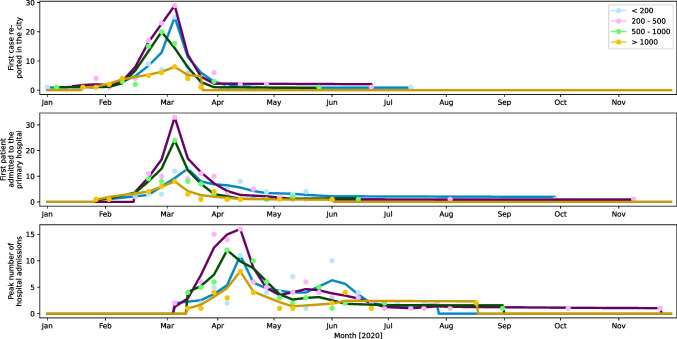
Same data stratified according to the hospital volume (data for hospitals with 1000–2000 beds and more than > 2000 beds was combined due to the limited number of hospitals within these groups)

For Figs. 1, 2, and 3, data were analysed and plotted in python 3.9 with in-house written python scripts using numpy (version 1.20.2), scipy (version 1.6.2), matplotlib (version 3.4.1), cartopy (version 0.18.0), and pandas (version 1.2.4). For Figs. 2 and 3, data were accumulated for each day data was reported, on. For clarity, all data was then fed through a low-pass Butterworth filter and the maximum of the data was added to the resulting curve. The smoothed data was then plotted as connected line segments.

## Results

A total of 527 surgeons from 439 hospitals in 64 countries submitted data on their practices and handling of the pandemic. Figure 1 gives an overview of the geographical distribution of hospitals included in this study. Table 1 shows the baseline data of the participating surgeons and centres.

**Table 1:** Data on the participating surgeons and centres

Individual surgeons’ data	
Years of (surgical/endoscopic) experience (mean ± SD)	12.58 ± 6.95
Case load	
< 500 500–999 1000–5000 > 5000	127 (24.1%) 84 (15.9%) 203 (38.5%) 48 (9.1%)
Hospital data	
Type of bariatric practice	
Private practice Government-funded practice Combination of both	210 (39.8%) 96 (18.2%) 157 (29.8)
Credentials primary hospital	
District general hospital Teaching hospital University hospital	196 (37.2%) 99 (18.8%) 167 (31.7%)
Total number of beds at primary hospital	
< 200 200–500 500–1000 1000–2000 > 2000	165 (31.3%) 146 (27.7%) 99 (18.8%) 40 (7.6%) 11 (2.1%)

*SD* standard deviation

Complete data with regards to all-time points were available for 276 unique practices. Only this data was used when analysing the effect of hospital volume or type of bariatric practice over handling the COVID-19 pandemic. All data was found to be not normally distributed.

### Effect of Type of Bariatric Practice/Hospital Volume and Handling of the COVID-19 Pandemic

Figure 2 shows the timeline of the first case diagnosed in the city, the first case admitted to the primary hospital, and the peak number of hospital admissions according to the practice type. The majority of the practices reported that the first patient with COVID-19 in their city was diagnosed in the second week of March 2020, and the first patient with COVID-19 was admitted to their primary hospital in the same week. Two peaks in hospital admissions were noticed. The first peak was between 09/03/2020 and 12/05/2020 in 192 out of 276 (70%) practices. The second peak was between 12/05/2020 and 06/07/2020 in 69 out of 276 (25%) practices. Figure 3 shows the same data stratified according to the hospital volume (data for hospitals with 1000–2000 beds and more than > 2000 beds was combined due to the limited number of hospitals within these groups.

When comparing privately funded practices with government-funded or combination practices, there was relative shielding of the private practices, with only 49 out of 109 (45%) having a peak in hospital admissions at a similar time when government and combination practices had a peak (09/03/20–04/05/20; peak incidence–13/04/2020; Fig. 2). This was followed by a second peak mainly in the private practices in May/ June (11/05/2020–22/06/2020). This was seen for 50 out of 109 (46%) of the private practices.

Of 276 practices (43.1%), 119 noticed a change in their bariatric practice before the first case was diagnosed in that city. Hospitals stopped their bariatric activity at a mean of 5 days (± 27.96) before the first case of COVID-19 was diagnosed in the city. Bariatric activity in private and combination practices stopped earlier than in government-funded practices (7.11 ± 29.22 and 7.16 ± 32.01 vs 1.39 ± 17.80 days) before the first case of COVID-19 was diagnosed in the city.

**Fig. 4:**
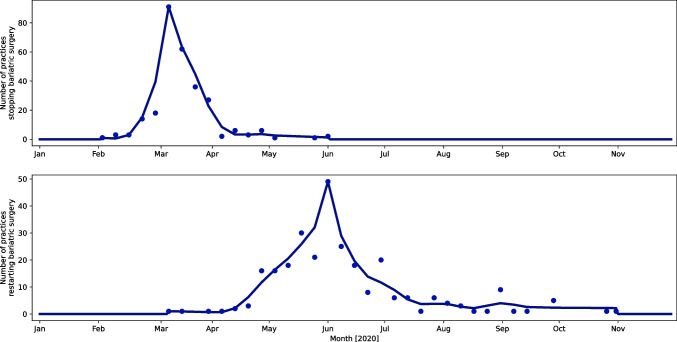
Timeline of the changes in bariatric activity due to the COVID-19 pandemic and when hospitals resumed bariatric surgical practice

Hospitals with a bed volume of < 200 beds had two distinct peaks of hospital admissions as opposed to hospitals with 200–500 beds, 500–1000 beds, or > 1000 beds. The first peak was reported between 09/03/20 and 04/05/20 with a peak incidence on 14/04/20, followed by a second peak between 11/05/20 and 22/06/20 with a peak incidence on 01/06/20 (Fig. 4). Thirty four of 72 (47%) practices with less than 200 beds reported a peak in hospital admissions during the first peak whilst 31 of 72 practices (44%) reported a peak during the second peak. Overall, the second peak in hospital admissions was less pronounced for larger hospitals. For hospitals with 200–500 beds, the second peak was noticed for 26 of 102 (25%) practices, followed by 15 of 70 (21%) for 500–1000 bedded hospitals and 10 of 32 (31%) for hospitals with > 1000 beds.

### Factors Affecting the Resumption of Bariatric Services

Figure 4 depicts a timeline of the changes in bariatric activity due to the COVID-19 pandemic and when hospitals resumed bariatric surgical practice. Out of 276 practices (90%), 248 reported changes in their bariatric practices between 23/02/20 and 06/04/20. Peaks in the resumption of elective bariatric surgery were seen between 20/04/20 and 27/7/2020 in 237 out of 276 practices (86%).

There was a significant difference in the time interval between the stoppage/reduction and restart of bariatric services between government-funded practices (97.1 ± 76.2 days), combination practices (84.4 ± 47.9 days), and private practices (58.5 ± 38.3 days) (*p* < 0.001; Kruskal–Wallis test). The time interval was significantly shorter in the private practices.

The resumption of services was again significantly in favour of smaller hospitals: 60.8 ± 38.9 days (< 200 beds) versus 76.8 ± 44.0 days (200–500 beds), 94.3 ± 80.3 days (500–1000 beds), 79.6 ± 51.8 days (1000–2000 beds), and 81.3 ± 30.5 days (> 2000 beds) (*p*-value = 0.032, Kruskal–Wallis test).

### Precautionary Measures

With regards to the management of patient flow, 266 (50.5%) surgeons reported that they had separate wards for elective and severe acute respiratory syndrome–coronavirus 2 (SARS-CoV-2)-positive patients, and 103 (19.5%) indicated that patients who tested positive were moved to separate sites. Thus overall, almost two thirds of the surgeons maintained segregation of elective and COVID-19 patients.

Regarding preoperative testing, 359 (68.1%) surgeons confirmed that patients had at least one preoperative Reverse Transcriptase–Polymerase Chain Reaction Test (RT-PCR) for SARS-CoV-2 infection. Similarly, a preoperative chest X-ray or Computed Tomography (CT) scan was advised by 148 (28.1%) and 108 (20.5%) surgeons, respectively. Preoperative self-isolation was advised by 269 (49.1%) of the surgeons.

The most popular precautionary measure amongst healthcare staff was wearing masks and self-recording of temperature and symptoms (Table 2). Staff members, nurses, surgeons, and non-clinical staff in 434 (82.4%), 435 (82.5%), and 393 (74.6%) of the cases, respectively, were advised to wear masks. Of the nurses, surgeons, and non-clinical staff, respectively, 338 (64.1%), 323 (61.3%), and 393 (56.4%) were advised to monitor their temperature and symptoms.

**Table 2:** Clinic and staff precautionary measures related to COVID-19

*N* = 527	
Masks to be warn in wards/clinics at all times	
Nursing staff Surgeons Non-clinical staff Not needed for any staff	434 (82.4%) 435 (82.5%) 393 (74.6%) 39 (7.4%)
Staff to be tested once for COVID-19 with PCR	
Nursing staff Surgeons Non-clinical staff Not needed for any staff	151 (28.7%) 153 (29.0%) 106 (20.1%) 288 (54.6%)
Staff to be tested weekly for COVID-19 with PCR	
Nursing staff Surgeons Non-clinical staff Not needed for any staff	63 (12.0%) 62 (11.8%) 37 (7.0%) 381 (72.3%)
Staff to be tested for COVID-19 antibodies (if PCR is positive	
Nursing staff Surgeons Non-clinical staff Not needed for any staff	154 (29.2%) 163 (30.9%) 113 (21.4%) 292 (55.4%)
Staff to maintain daily for symptoms and temperature monitoring	
Nursing staff Surgeons Non-clinical staff Not needed for any staff	338 (64.1%) 323 (61.3%) 297 (56.4%) 137 (26.0%)
What ‘eligibility to work’ protocols does your primary hospital use for staff?	
Antibody positive PCR negative PCR positive staff must be antibody positive Other No protocols	17 (3.2%) 193 (36.6%) 17 (3.2%) 82 (15.6%) 150 (28.5%)

*COVID-19* corona virus disease-19, *PCR* polymerase chain reaction

Of the surgeons, 270 (51.2%) reported that Personal Protection Equipment (PPE) kits were immediately available, and 81 (15.4%) said there was a delay in availability varying between 0 and 30 days after the publication of the guidelines of the World Health Organisation (WHO).

### Impact of COVID-19 Pandemic on Bariatric Practice

Four-hundred forty-eight out of 527 (85%) surgeons reported that the COVID-19 pandemic had decreased their elective bariatric surgical practice. Six (1.1%) surgeons had an increase in elective bariatric surgical practice, and 5 (0.9%) had no change. In five of the six cases where surgeons reported an increase in elective bariatric surgical practice, the increase was attributed to the patients seeking weight loss for protection from COVID-19. One of the six was due to the re-allocation of elective surgery to the so-called COVID-19-free hospital. Four-hundred (75.9%) surgeons reported that elective bariatric surgical practice ceased completely at some time point.

Around 40% of the surgeons operated with a reduced capacity, 22% gave priority to long waiters, 15.4% gave priority to uncontrolled diabetics, and 7.6% prioritised patients requiring organ transplantation (Table 3). Only a minority continued without any restrictions.

**Table 3:** Policy of bariatric practice on resumption of service

*N* = 527	
Long waiters given priority	116 (22.0%)
Reduced number of bariatric procedures allowed	211 (40%)
Patients with uncontrolled diabetes given priority	81 (15.4%)
Patients requiring organ transplantation were given priority	40 (7.6%)
No restrictions	111 (21.1%)

*COVID-19* corona virus disease-19

## Discussion

The present study has provided real-world data regarding the process of recovery of BMS services following the COVID-19 pandemic. The study demonstrates that the majority of hospitals stopped BMS operations before the first patient was diagnosed with COVID-19 in that geographical area and that this suspension of services occurred more swiftly in privately funded units than in government institutions.

This study found that smaller hospitals (with less than 200 beds) were able to restart BMS services more rapidly (time to BMS restart 60.8 ± 38.9 days) than larger institutions (over 2000 beds) (81.3 ± 30.5 days) (*p* = 0.032). The reasons for this are likely to be multifactorial. These hospitals may have been less severely affected during the initial COVID-19 wave. Larger hospitals may also have had a greater burden of critically unwell patients requiring more specialist services. These patients are likely to have had more prolonged admissions during their COVID-19 treatment [12].

Another critical aspect of elective surgery recovery following COVID-19 has been the necessity to segregate patients on elective ‘green’ pathways where patients are specifically screened before admission to prevent the transmission of COVID-19. In many instances, this has been achieved through the utilisation of separate hospital sites for elective patients. This was also identified in the current study with the majority of surgeons utilising some form of patient segregation and almost 20% treating elective patients on a separate hospital site where COVID-19 patients were being managed. This development of separate hospital sites for elective surgery may also provide a further potential explanation of the finding that smaller units were able to resume BMS at earlier stages as these may have been selected as the potential green site for these services. It is to be noted that these green pathways in this study were formulated between May and October 2020, before their value became more widely reported [13].

BMS patients generally do not require a large number of intensive care or high-dependency unit beds compared to other specialities such as cancer or transplant surgery [14]. The majority of specialist equipment (such as laparoscopic equipment, appropriate operating tables, and ward-based patient care items) are potentially transferable between hospital sites. This makes BMS a very adaptable surgical specialty that can be relatively self-contained and is well suited to be transferred to an alternative hospital site as and when necessary.

A further significant finding in this dataset was that privately funded institutions were able to resume BMS services more swiftly than government institutions. Again, the reasons for this are likely to be multifactorial and may vary globally. Many private institutions were also smaller (just under half of the private hospitals had less than 200 beds) and may have been influenced by the factors described above. Furthermore, in many healthcare systems such privately funded hospitals would not have been treating large numbers of COVID-19 patients. During the recovery phase many countries saw close collaboration between private providers and government institutions. In some areas, these private units were specifically utilised as the ‘green’ sites described above to treat publicly funded patients that would otherwise have been treated in government institutions [15, 16]. This form of collaboration allowed for elective surgery to resume much more rapidly in many areas than relying upon government institutions alone.

During the recovery phase of the COVID-19 pandemic, many surgeons were required to prioritise patients to facilitate the appropriate resource utilisation. Many surgeons in the present study (40%) reported that they were operating with reduced capacity compared to the pre-pandemic level. There was some variation in priority groups with some units focussing on long-waiting patients (22.0%), whilst a smaller number prioritised those with uncontrolled diabetes (15.4%) or those awaiting organ transplant (7.6%). This is interesting and seems to be at odds with the guidance provided during the early stages of the pandemic [4, 17].

Aside from the direct effect on surgical services, the COVID-19 pandemic has seen the need for widespread lockdown restrictions which have had a profound negative effect on multiple aspects of health-related behaviours such as eating habits and physical activity, and other barriers to weight management [18]. There were also major changes concerning how the whole range of weight management services was delivered including access to services and the increased use of telemedicine for care delivery [19]. These changes were welcomed by patients and can improve efficiency for healthcare professionals, and are therefore likely to become part of the long-term care model for future weight management services [20]. Such adaptations of services are likely to be maintained even after the pandemic. However, data regarding the effectiveness of such strategies have been controversial [21], although the majority of studies have demonstrated effective results following BMS during the COVID-19 pandemic [22, 23].

Although the present study has provided an overview of the effect of the initial stages COVID-19 pandemic on BMS services the period of data collection ended in October 2020. As the COVID-19 pandemic has continued to progress it has to date been unclear how this has influenced the delivery of BMS services in 2021 and 2022. Data recently published from the National Obesity Audit in England have demonstrated that although the number of cases performed between April 2021 and April 2022 (*n* = 4440) has improved compared to 2020/2021 (*n* = 1854) this has still not reached pre-pandemic rates of BMS (2018/2019, *n* = 6779) [24]. Data from the Metabolic and Bariatric Surgery Accreditation and Quality Improvement Programme (MBSAQIP) in the United States has also confirmed a reduction in BMS during 2020 of approximately 22.5% [25], but data regarding the recovery of services in 2021 and beyond is awaited. In the future, it will also be necessary to establish if there has been any increase in referrals for BMS which may have been triggered due to concerns regarding the effect of severe obesity on outcomes following SARS-CoV-2 infection [26, 27].

Significant strengths of the present study are that it provides large-scale real-world data on the recovery of BMS services during the COVID-19 pandemic globally. Despite this, several important limitations must be considered when interpreting these results. Data were self-reported and not externally validated, therefore is reliant upon the accuracy of data input by individual collaborators. Due to the global nature of this study, there was significant variation in healthcare structure across participating centres (particularly the relationship between government and privately funded institutions). Data regarding screening and precautionary measures for COVID-19 was collected before the widespread introduction of rapid lateral flow tests for this purpose, and therefore data regarding their utilisation was not collected. The present study was also conceived and executed before the widespread utilisation of vaccination against SARS-CoV-2 for the general population so it has not been possible to assess the influence of the vaccination programme on BMS.

## Conclusion

The present study has provided global, real-world data regarding the recovery of BMS services during the COVID-19 pandemic.

## Supplementary Information

ESM 1 (PDF 63.9 KB)

## Data Availability

The data used to support the findings of this study can be released upon request.
