# Supplementary material for: Handling of the Covid-19 Pandemic and Its Effects on Bariatric Surgical Practice: Analysis of GENEVA Study Database
Source: Obes Surg. 2022 Oct 25;32(12):3908–21. doi: 10.1007/s11695-022-06267-7 (PMC9592878; doi:10.1007/s11695-022-06267-7)
Supplement: Supplementary file 1 — (PDF 63.9 KB) [file 11695_2022_6267_MOESM1_ESM.pdf]

Bariatric Practice

This form asks about your primary practice and the effect COVID19 has had on your Bariatric surgery. There are 28 questions (excluding branching/associated questions). It is envisaged that it should take under 30 minutes to complete and you will only have to complete it once.

Thank you for taking this time to complete the form

About You (3 Questions)

1 What country do you work in?

- ◯ Afghanistan
- ◯ Albania
- ◯ Algeria
- ◯ Andorra
- ◯ Angola
- ◯ Antigua and Barbuda
- ◯ Argentina
- ◯ Armenia
- ◯ Australia
- ◯ Austria
- ◯ Azerbaijan
- ◯ Bahamas
- ◯ Bahrain
- ◯ Bangladesh
- ◯ Barbados
- ◯ Belarus
- ◯ Belgium
- ◯ Belize
- ◯ Benin
- ◯ Bhutan
- ◯ Bolivia
- ◯ Bosnia and Herzegovina
- ◯ Botswana
- ◯ Brazil
- ◯ Brunei
- ◯ Bulgaria
- ◯ Burkina Faso
- ◯ Burundi
- ◯ Cabo Verde
- ◯ Cambodia
- ◯ Cameroon
- ◯ Canada
- ◯ Central African Republic (CAR)
- ◯ Chad
- ◯ Chile
- ◯ China
- ◯ Colombia
- ◯ Comoros
- ◯ Congo, Democratic Republic of the
- ◯ Congo, Republic of the
- ◯ Costa Rica
- ◯ Cote d'Ivoire
- ◯ Croatia
- ◯ Cuba
- ◯ Cyprus
- ◯ Czechia
- ◯ Denmark
- ◯ Djibouti
- ◯ Dominica
- ◯ Dominican Republic
- ◯ Ecuador
- ◯ Egypt
- ◯ El Salvador
- ◯ Equatorial Guinea
- ◯ Eritrea
- ◯ Estonia
- ◯ Eswatini (formerly Swaziland)
- ◯ Ethiopia
- ◯ Fiji
- ◯ Finland
- ◯ France
- ◯ Gabon
- ◯ Gambia
- ◯ Georgia
- ◯ Germany
- ◯ Ghana
- ◯ Greece
- ◯ Grenada
- ◯ Guatemala

◯ Guinea
◯ Guinea-Bissau
◯ Guyana
◯ Haiti
◯ Honduras
◯ Hungary
◯ Iceland
◯ India
◯ Indonesia
◯ Iran
◯ Iraq
◯ Ireland
◯ Israel
◯ Italy
◯ Jamaica
◯ Japan
◯ Jordan
◯ Kazakhstan
◯ Kenya
◯ Kiribati
◯ Kosovo
◯ Kuwait
◯ Kyrgyzstan
◯ Laos
◯ Latvia
◯ Lebanon
◯ Lesotho
◯ Liberia
◯ Libya
◯ Liechtenstein
◯ Lithuania
◯ Luxembourg
◯ Madagascar
◯ Malawi
◯ Malaysia
◯ Maldives
◯ Mali
◯ Malta
◯ Marshall Islands
◯ Mauritania
◯ Mauritius
◯ Mexico
◯ Micronesia
◯ Moldova
◯ Monaco
◯ Mongolia
◯ Montenegro
◯ Morocco
◯ Mozambique
◯ Myanmar (formerly Burma)
◯ Namibia
◯ Nauru
◯ Nepal
◯ Netherlands
◯ New Zealand
◯ Nicaragua
◯ Niger
◯ Nigeria
◯ North Korea
◯ North Macedonia (formerly Macedonia)
◯ Norway
◯ Oman
◯ Pakistan
◯ Palau
◯ Palestine
◯ Panama
◯ Papua New Guinea
◯ Paraguay
◯ Peru
◯ Philippines
◯ Poland

◯ Portugal
◯ Qatar
◯ Romania
◯ Russia
◯ Rwanda
◯ Saint Kitts and Nevis
◯ Saint Lucia
◯ Saint Vincent and the Grenadines
◯ Samoa
◯ San Marino
◯ Sao Tome and Principe
◯ Saudi Arabia
◯ Senegal
◯ Serbia
◯ Seychelles
◯ Sierra Leone
◯ Singapore
◯ Slovakia
◯ Slovenia
◯ Solomon Islands
◯ Somalia
◯ South Africa
◯ South Korea
◯ South Sudan
◯ Spain
◯ Sri Lanka
◯ Sudan
◯ Suriname
◯ Sweden
◯ Switzerland
◯ Syria
◯ Taiwan
◯ Tajikistan
◯ Tanzania
◯ Thailand
◯ Timor-Leste
◯ Togo
◯ Tonga
◯ Trinidad and Tobago
◯ Tunisia
◯ Turkey
◯ Turkmenistan
◯ Tuvalu
◯ Uganda
◯ Ukraine
◯ United Arab Emirates (UAE)
◯ United Kingdom (UK)
◯ United States of America (USA)
◯ Uruguay
◯ Uzbekistan
◯ Vanuatu
◯ Vatican City (Holy See)
◯ Venezuela
◯ Vietnam
◯ Yemen
◯ Zambia
◯ Zimbabwe
◯ Other

Country Other : Please enter your country ________________________________

Please allow location services for automated latitude - (click on update - location services must be turned on) ________________________________

	Please allow location services for automated longitude - (click on update - location services must be turned on)	__________________
2	Please enter your highest qualification (for the purposes of publication)	__________________
3	What type of bariatric practices are you affiliated with?	◯ Private practice only<br>◯ Government-funded practice only<br>◯ Combination of both

About Your primary hospital (6 Questions)
(Your primary hospital is where you do the majority of your bariatric procedures)

4	Name of Primary Hospital (for publication purposes)	__________________
5	Which city is your primary bariatric practice located in?	__________________
6	What is the total number of beds at your primary hospital?	◯ < 200<br>◯ 200 - 500<br>◯ 500 - 1000<br>◯ 1000 - 2000<br>◯ >2000
7	Which of the following apply to your primary hospital ?	◯ District general hospital<br>◯ Teaching hospital<br>◯ University Hospital (one which is affiliated with a medical school/ college)
8	How long have you been practicing bariatric surgery/ bariatric endoscopy (in years)?	__________________
9	How many bariatric procedures (surgical and endoscopic) have you performed? (Estimate)	◯ < 500<br>◯ 500-999<br>◯ 1000-5000<br>◯ >5000

COVID 19 and your Primary Hosptial (10 Questions)

Please be as accurate as possible with the following questions, as dates will be used to calculate the chronology/ time-line of COVID19 in your area/ hospital and its impact on bariatric practice.

10	When was the first case of COVID-19 (Corona Virus) reported in your city? (please give your best estimate if you are not completely certain)	__________________<br>((D-M-Y))
11	Were any patients with COVID-19 admitted to your primary hospital?	◯ Yes<br>◯ No
	When was the first patient with COVID-19 admitted to your primary hospital? (please give your best estimate if you are not completely certain)	__________________<br>((D-M-Y))

What was the maximum number of COVID-19 patients treated in your hospital at any one time including ITU admissions? - (please give your best estimate if you are not completely certain)

____

When was the peak of COVID-19 in your hospital in terms of number of admissions? (please give your best estimate if you are not completely certain)

____
((D-M-Y))

12 What impact has COVID19 had on you elective (routine or planned) bariatric surgical practice?

◯ Decreased ◯ No Change ◯ Increased

What caused the increase in your elective surgeries?

Why was there no change in your elective surgery load?

Was COVID19 the only reason for the decrease in elective bariatric surgery?

When did the change in elective bariatric surgery volume begin? (please give your best estimate if you are not completely certain)

____
((D-M-Y))

13 Did your elective bariatric practice cease completely at any point?

◯ Yes ◯ No

When did elective bariatric surgery stop? (please give your best estimate if you are not completely certain)

____
((D-M-Y))

When did bariatric surgery restart or when do you plan to restart? (please give your best estimate if you are not completely certain)

____
((D-M-Y))

14 What restrictions are there to bariatric practice for the duration of COVID in your primary hospital?

- ☐ Long waiters given priority
- ☐ Reduced number of bariatric procedures allowed
- ☐ Patients with uncontrolled diabetes given priority
- ☐ Patients requiring organ transplantation given priority
- ☐ No restrictions
- ☐ Other

Other restricitons

15 What is your balance of priority for the duration of COVID in these patients?

Low BMI, No-comorbidities	In order of waiting list	High BMI, mutiple and uncontrolled co-morbidities

(Place a mark on the scale above)

16 How many COVID+ patients do you currently have in your hospital including ITU admissions? (please give your best estimate if you are not completely certain)

____

17 After restarting bariatric surgery, will your hospital continue to treat COVID19 patients? ◯ Yes ◯ No

Where are/will the COVID+ patients (be) treated? ________________________________

What precautions are being/will be taken to prevent elective bariatric surgery patients coming in contact with COVID-19 patients at your facility?

- ☐ Separate wards (elective and COVID positive)
- ☐ Separate theatres
- ☐ COVID +ve patients will be moved to a new site
- ☐ None of the above
- ☐ Other

Other precautions ________________________________

18 Which of the following form part of your preoperative screening protocol at the start of bariatric surgery?

- ☐ Two COVID19 PCR tests pre-operatively (approx. 3 and 2 days prior to surgery)
- ☐ One COVID19 PCR test preoperatively
- ☐ One COVID19 antibody test preoperatively
- ☐ Preoperative Chest X-ray
- ☐ Preoperative Chest CT Scan
- ☐ None of the above
- ☐ Other

Other Preoperative Surgery Protocol ________________________________

19 Which mandatory self isolation process will apply to patients?

- ◯ One week pre-op
- ◯ Two weeks pre-op
- ◯ One week post-op
- ◯ Two week post-op
- ◯ Other
- ◯ No mandatory isolation

isolation: other

____

Is patient allowed to be with family member(s) or carer(s) during self-isolation?

- ◯ Yes
- ◯ No
- ◯ Not Applicable

COVID specific precautions for hospital staff (8 questions)

		Nursing staff	Surgeons	Non-clinical staff (cleaners/porters)	Not needed for any staff
20	Masks to be worn in wards/ clinics at all times	☐	☐	☐	☐
21	Staff to be tested once for PCR	☐	☐	☐	☐
22	Staff to be tested weekly for PCR	☐	☐	☐	☐
23	Staff to be tested for antibody (if positive for PCR)	☐	☐	☐	☐
24	Staff to maintain diary for symptoms and temperature monitoring	☐	☐	☐	☐

25 What "eligibility to work" protocols does your primary hospital use for staff?

- ◯ PCR negative
- ◯ PCR positive staff must be antibody positive
- ◯ Antibody positive
- ◯ No protocols
- ◯ Other

Eligibility to work protocols other

26 What was the time lag between WHO guidelines for use of PPE during surgery and endoscopy, and appropriate PPE equipment being made available at your hospital?

- ◯ PPE equipment was available immediately after guidelines were issued.
- ◯ There was a time lag of 0 - 10 days.
- ◯ There was a time lag of 10 - 20 days.
- ◯ There was a time lag of 20 - 40 days.
- ◯ All appropriate PPE equipment is still not available at my hospital.
- ◯ Other

Other

____

27 In which cases do you use full PPE (including a FFP3 or N5 mask) in theatres? Please select all that apply.

- ☐ Cases where PCR/ antibody status not known
- ☐ Cases where PCR positive but antibody negative
- ☐ All cases
- ☐ No cases
- ☐ Other

other cases using full PPE (including a FFP3 or N5 mask) in theatres

28 With regards to endoscopic bariatric procedures at your primary hospital, which of the following will apply to the use of PPE ?

- ◯ Full PPE (including FFP3/N95 mask) for all endoscopic procedures
- ◯ Full PPE only for cases where PCR/ antibody status not known
- ◯ Full PPE for cases that are PCR postive, antibody negative
- ◯ Endoscopic procedures will be performed without full PPE
- ◯ Not applicable
- ◯ Others

other uses of PPE

Would you like to collaborate with us in future studies based on a collaborative model of authorship?

- ◯ Yes
- ◯ No
